# Great Abilities of *Shinella zoogloeoides* Strain from a Landfarming Soil for Crude Oil Degradation and a Synergy Model for Alginate-Bead-Entrapped Consortium Efficiency

**DOI:** 10.3390/microorganisms10071361

**Published:** 2022-07-06

**Authors:** Emerance Jessica Claire D’Assise Goma-Tchimbakala, Ilaria Pietrini, Federica Dal Bello, Joseph Goma-Tchimbakala, Stefano Lo Russo, Stefano Paolo Corgnati

**Affiliations:** 1 Energy Center Lab, Department of Energy, Politecnico di Torino, 10138 Torino, Italy; stefano.corgnati@polito.it; 2 Institut National de Recherche en Sciences Exactes et Naturelles (IRSEN), Brazzaville P.O. Box 2400, Congo; goma.tchimbakala@gmail.com; 3 Eni R&D, Environmental and Biological Laboratories, Eni SpA, 20097 San Donato Milanese, Italy; ilaria.pietrini@eni.com; 4 Department of Molecular Biotechnology and Health Sciences, Università degli Studi di Torino, 10125 Torino, Italy; federica.dalbello@unito.it; 5 Ecole Nationale Supérieure d’Agronomie et de Foresterie, Université Marien Ngouabi, Brazzaville P.O. Box 69, Congo; 6 Department of Environment, Land and Infrastructure Engineering, Politecnico di Torino, 10138 Torino, Italy; stefano.lorusso@polito.it

**Keywords:** *Shinella zoogloeoides*, hydrocarbon bioremediation, GC-MS, alginate bead entrapment, metagenomics

## Abstract

Oil contamination is of great concern worldwide and needs to be properly addressed. The present work aimed to contribute to the development of bacterial consortia for oil recovery. We investigated the community structure of a landfarming-treated soil (LF2) by metagenomics to unravel the presence of hydrocarbon degraders. Moreover, we isolated *Shinella zoogloeoides* LFG9 and *Bacillus swezeyi* LFS15 from LF2 and combined them with *Pseudomonas guguanensis* SGPP2 isolated from an auto mechanic workshop soil to form the mixed consortium COG1. Bacterial isolates were tested for biosurfactant production. Additionally, the bioremediation potential of COG1 was studied as free and entrapped consortia by gas chromatography-mass spectrometry, in comparison to the single strains. Results revealed the presence of Actinobacteria (66.11%), Proteobacteria (32.21%), Gammaproteobacteria (5.39%), Actinomycetales (65.15%), Burkholderiales (13.92%), and *Mycobacterium* (32.22%) taxa, indicating the presence of hydrocarbon degraders in soil LF2. All three isolated strains were biosurfactant producers capable of degrading crude oil components within 14 days. However, *Shinella zoogloeoides* LFG9 performed best and was retained as candidate for further bioremediation investigation. In addition, COG1 performed better when immobilized, with entrapment effectiveness manifested by increased fatty acids and aromatic compound degradation. Attempt to improve crude oil biodegradation by adding surfactants failed as sodium dodecyl sulfate restrained the immobilized consortium performance.

## 1. Introduction

The intensive exploitation of oil resources to supply energy demands and human activities is of great concern. Indeed, this practice might be accompanied with loss, leaks, or spills during the refinement process, storage, or transportation, leading to the accidental contamination of the environment [1,2].

Hydrocarbons from petroleum wastes are recalcitrant and hazardous compounds with acute and chronic effects [1,3], necessitating their elimination from terrestrial ecosystems. For this purpose, different techniques for hydrocarbon removal have been developed. The conventional methods, involving the use of heat or chemical and physical processes are costly and eco-unfriendly. Therefore, they have been marginalized to the benefit of biological techniques employing living organisms [4]. Especially, the use of hydrocarbon-degrading micro-organisms that have been enriched and adapted to contaminated sites has been proposed over the last few years as a sustainable approach to remediate oil-contaminated areas [2,5,6]. Remediation can be conducted as a bioaugmentation process, corresponding to the enhancement of the indigenous microbial community by adding selected strains with known hydrocarbon biodegradative abilities in the contaminated matrix [7]. Bioaugmentation is particularly efficient when a mixture of micro-organisms is employed. Indeed, in nature, contaminant degradation is completed through the synergetic actions of multiple micro-organisms. Mixed communities present broader enzymatic and catabolic abilities allowing the degradation of more diverse petroleum fractions [6,8].

Oil remediation can also be improved by the application of surface-active agents. Surfactants reduce the surface tension at the oil–water interface, thus encapsulating oil in microdroplets and consequently making it more bioavailable [4]. Micro-organisms, including bacterial genera *Bacillus*, *Pseudomonas*, *Rhodococcus*, *Acinetobacter*, *Corynebacterium*, and *Nocardia* [6,9,10,11], are able to synthesize surface active agents called biosurfactants from cheap substrates, such as industrial wastes, or from carbon sources, such as oils [4,11]. Biosurfactants are amphiphilic molecules with hydrophilic and hydrophobic moieties that optimize the microbial uptake of petroleum hydrocarbons by increasing the contact area with the contaminants. In addition, their mobility is also increased to facilitate their translocation across the cell membrane and their subsequent degradation [11]. Biosurfactants are biodegradable, environmentally friendly, and highly selective, with minimal toxicity unlike their chemical counterparts. They are therefore applicable in a variety of industries and are especially recommended for oil removal in the environment [11]. In this regard, biosurfactant production has been reported to be an effective means to improve petroleum bioremediation [3,7,12]. The use of hydrocarbon-degrading bacteria with the ability to produce biosurfactants is therefore advantageous for bioremediation application. However, this combination is not necessarily sufficient to ensure successful bioaugmentation. The micro-organisms introduced must be able to resist not only harsh environmental conditions but also possible competitive interactions with the indigenous micro-organisms [1,13]. In this context, the provision of a niche ensuring protection as well as long-lasting survival and efficiency of the inoculum is recommended. A largely approved approach to achieve this goal is the immobilization of the consortium on a support. The carriers can be of different natures, including alginate, chitosan, polyvinyl alcohol, guar gum, and wheat bran, and immobilization can be performed by adsorption, aggregation, confinement, and entrapment [1,8]. One of the most common immobilization techniques for hydrocarbon removal is the entrapment of microbial consortia in alginate beads. The technique is eco-friendly, easy to perform, and has been shown to be suitable for oil recovery in polluted soil [1].

This report is part of a broad study aiming to develop a bioremediation strategy test model employing bacterial consortia for oil recovery. In this perspective, the present contribution has dual objectives. Firstly, we investigated the effects of alginate bead entrapment on hydrocarbon degradation efficiency of a consortium of bacteria isolated from soils in the Republic of Congo. This is the very first time such a study has been carried out on bacterial isolates from polluted soils originating from Congo. Indeed, although research on microbial degradation of hydrocarbons has expanded in the country over the last few years [9,14], there is no study on microbial entrapment for oil recovery. Usually, in remediation studies, strains are isolated from polluted soils that have not been submitted to any type of remediation. Here, we associated strains from an auto mechanic workshop and a landfarming-treated soil to form the consortium. Beforehand, the microbial community structure of the landfarming-treated soil was investigated by shotgun metagenomics to detect the presence of hydrocarbon-degrading taxa. Secondly, the present study aimed at selecting best candidates for further development of the test model strategy. In this regard, individual strains’ abilities to degrade crude oil and produce biosurfactants were explored. In this context, for the first time, *Shinella zoogloeoides* has been tested for its abilities in oil recovery by means of biosurfactant production, ultimately revealing the isolated strain *Shinella zoogloeoides* LFG9 as the best candidate for hydrocarbon bioremediation among the bacterial consortium members.

## 2. Materials and Methods

### 2.1. Illumina Sequencing and Processing of Assembled Genomes

The LF2 soil sample used in this study was collected after a landfarming experiment and provided by a local company in Congo. The soil characteristics are presented in Table 1.

The soil metagenome was sequenced and processed at Mr DNA laboratory, Texas, in the following process. DNA was extracted from 250 mg of the soil sample using the PowerSoil^®^ DNA Isolation Kit (Qiagen, Hilden, Germany). The concentration of DNA was evaluated using the Qubit^®^ dsDNA HS Assay Kit (Life Technologies, Carlsbad, CA, USA). The libraries were prepared from 20–50 ng of DNA using Nextera DNA Sample preparation kit (Illumina, San Diego, CA, USA), following the manufacturer’s user guide. The sample underwent the simultaneous fragmentation and addition of adapter sequences. These adapters were utilized during a limited-cycle (five cycles) PCR in which unique indices were added to the sample. Following the libraries’ preparation, the final concentrations of the libraries were measured using the Qubit^®^ dsDNA HS Assay Kit (Life Technologies), and the average library size was determined using the Agilent 2100 Bioanalyzer (Agilent Technologies, Santa Clara, CA, USA). The libraries were pooled and diluted (to 14.0 pM) and paired-end sequenced for 300 cycles using the HiSeq system (Illumina). The obtained high-quality (i.e., quality score > 35) reads were processed using Galaxy instances [15] (https://galaxyproject.org/ (accessed on October 2021). The paired-end reads were assembled with MEGAHIT version 1.1.3.5 (accessed on October 2021) [16] considering the k-mer values 39, 59, 79, 99, 119, 141, and 500 as thresholds for contig length. The reads were then mapped against the assembled genomes in Bowtie2 version 2.3.4.3 (accessed on October 2021) [17]. Following the filtration of potential duplicates and chimeric sequences, the obtained contigs were used as final results for taxonomic affiliation with MetaPhlAn2 v2.6.0.0 (accessed on October 2021) [18], considering markers at a minimum total nucleotide length of 500.

### 2.2. Bacterial Isolation and Enrichment Microcosms

The cultivable aerobic microbial community was isolated by adding 1 g of LF2 to 9 mL of sterile physiologic water. The mixture was left to rest for 6 h and then was serially diluted 10 times. Then, 100 µL of the dilutions were poured on Plate Count Agar (PCA) medium. The plates were incubated at 37 °C for 24 h.

In order to isolate hydrocarbon-degrading bacteria, an enrichment technique was used. Enrichment allows the selective growth of the micro-organisms of interest by choosing specific sources of carbon. In this study, diesel was the carbon source selected and used only for the enrichment phase. Instead, the degradation tests were performed using crude oil. Enriched microcosm cultures were prepared following a slightly modified technique by Ref. [19]. A total of 10 g of LF2 were inoculated in 90 mL of sterile Bushnell Haas (BH) medium (g/L: 0.2 MgSO_4_, 0.02 CaCl_2_, 1 KH_2_PO_4_, 1 K_2_HPO_4_, 1 (NH_4_)_2_SO_4_, 0.05 FeCl_3_, pH 7) in 250 mL flasks. The flasks were then incubated on a rotary shaker (250 rpm) at room temperature for 3 days. After this period, 10 mL of the culture suspension were inoculated in fresh sterile BH medium supplemented with 1% diesel. The mixture was incubated at 30 °C under agitation (150 rpm). After 7 days, 10 mL of the culture was transferred to another fresh BH medium and incubated in the same conditions with diesel as a hydrocarbon substrate. The experiment was renewed, making the enrichment last a total period of 21 days (3 cycles of 7 days each). The last enrichment culture was finally plated on PCA medium.

Another soil sample, SGP, contaminated with diesel and originating from an auto mechanic workshop, was included in this study in order to build a mixed consortium. Bacterial strains from SGP were isolated using the enrichment technique as performed for LF2. Instead of PCA, a specific cetrimide medium for the isolation of *Pseudomonas* species was used for plating the enrichment culture. *Pseudomonas* species are known to be among the best performers regarding petroleum hydrocarbon degradation [3].

The obtained bacterial isolates were then purified by successive subculturing.

### 2.3. DNA Extraction and Identification of Bacterial Strains

Genomic DNA was extracted from pure bacterial strains using Maxwell 16 (Promega, Madison, WI, USA), according to the manufacturer’s instructions. Bacteria were identified by sequencing the 16S rDNA using a pair of primers, 27-Forward (5′AGAGTTTGATCMTGGCTCAG-3′) and 1492-Reverse (5′-TAC GGY TAC CTT GTT ACG ACTT-3′), to amplify the V3 and V4 regions. The amplification program was set up as follows: initial denaturation at 95 °C for 1 s, 25 cycles of denaturation at 95 °C for 30 s, hybridization at 51 °C for 30 s, and 60 °C elongation for 4 min. 16S rRNA amplicons were then sequenced with SeqStudio Genetic Analyzer (ThermoFisher Scientific, Breda, The Netherlands). The taxonomic affiliation of bacteria was performed by Blast (Basic Local Alignment Search Tool/https://blast.ncbi.nlm.nih.gov/Blast.cgi (accessed on October 2021)) analysis against Genbank database.

### 2.4. Biosurfactant Production Assays

The molecular identification of bacterial isolates revealed the presence of potentially pathogenic strains. Those strains were therefore discarded. The four nonpathogenic strains were tested for their ability to produce biosurfactants in the presence of crude oil and hexadecane (Sigma-Aldrich, Milan, Italy).

#### 2.4.1. Oil-Coated Agar

Following the principle of the technique used by [20], bacteria were streaked on LB agar plates supplemented with crude oil (30 µL) and incubated for 5 days at 32 °C. The biosurfactant activity was detected by the presence of an emulsification halo around the culture growth.

#### 2.4.2. Emulsification Test

Using a UV-visible spectrophotometer (Jenway 7305, France), 1.5 mL of standardized pre-cultures (OD = 1 ± 0.2 at 600 nm) was inoculated in sterile liquid LB medium supplemented with 1% crude oil and incubated at 30 °C/150 rpm for 5 days. After incubation, the supernatant was collected by centrifugation (10,000 rpm for 15 min). A total of 2 mL of supernatant was mixed with equal volumes of crude oil and hexadecane in test tubes. The mixture was vortexed at high speed for 2 min and left at room temperature for 24 h [6]. The emulsification activity after 24 h (E_24_) was then determined according to the formula:

E_24_ = (He/Ht) × 100 (1)

where He is the height of the emulsification layer and Ht is the total height of the mixture. The experiment was conducted in triplicate.

### 2.5. Building the Bacterial Consortium

Three bacterial strains (2 from LF2 and 1 from SGP) were chosen to build a mixed consortium. The efficiency of the bacterial consortium for hydrocarbon degradation was tested using crude oil as the sole source of carbon in microcosm conditions.

#### 2.5.1. Choice of Temperature for Biodegradation Tests Setup

In order to determine the best temperature to set up crude oil biodegradation tests, the growth of the three bacterial strains chosen to build the consortium was assessed at 30 °C and 37 °C.

For each bacterial strain, a pre-culture was prepared. Bacteria were inoculated in 10 mL of LB medium and incubated for 48 h at 150 rpm. The pre-cultures were standardized at OD ± 1 at 600 nm. An amount of 1 mL of each pre-culture was then incubated in 100 mL of fresh sterile LB and incubated under agitation (150 rpm) at the two chosen temperatures (30° and 37 °C) for 6 days. The initial and final optical densities were measured. Growth was determined as the difference between the two values. The experiment was conducted in duplicate.

#### 2.5.2. Biodegradation Tests Setup

Pre-cultures of each bacterial strain were prepared in liquid LB medium and standardized to an optical density of OD = 1 ± 0.2 at 600 nm. The pellet was recovered from the pre-cultures by centrifugation at 10,000 rpm for 10 min and washed twice with sterile distilled water (4500 rpm for 15 min). Afterwards, for each strain, a seed inoculum was prepared as follows: The pellet was inoculated in 50 mL of mineral salt medium MSM (g/L:1.8 K_2_HPO_4_, 1 NH_4_Cl, 0.2 MgSO_4_.7H_2_O, 0.1 NaCl, 0.01 FeSO_4_.7H_2_O, pH 7) contained in 100 mL flasks and supplemented with 1% (*v*/*v*) crude oil and incubated at 37 °C for 48 h under agitation at 200 rpm. The consortium was finally prepared by inoculating equal volumes (0.5 mL) of seed inoculums in fresh sterile MSM medium prepared as earlier. The final 50 mL volume containing 1% crude oil was cultured in a shaking incubator at 37 °C at 150 rpm. The incubation was performed for 5 months. A control flask containing MSM medium supplemented with 1% crude oil but without bacterial addition was incubated at the same time and in the same conditions. Single strains were also inoculated following the same procedure in order to assess their individual efficiency during a 14-day incubation period.

### 2.6. Entrapment of the Bacterial Consortium

The entrapment of the bacterial consortium in calcium alginate was performed following a technique described in [21] with some modifications. The pellet from bacteria pre-cultures was recovered and washed as mentioned above. Cell suspensions were prepared by inoculating the pellet in sterile distilled water. The cell suspensions were then adjusted to OD = 1.5 at 600 nm. The three bacterial suspensions were mixed in equal volumes under continuous agitation by magnetic stirring. A 4% sodium alginate solution was also prepared and sterilized. The sodium alginate solution was added to the bacterial mixture in equal volumes and mixed for 30 min in order to obtain a final sodium alginate solution of 2% containing the bacterial biomass. Finally, the obtained mix was carefully extruded into a 0.2 M calcium chloride solution through a 5 mL syringe. The formed calcium alginate beads were left to strengthen for 1 h at room temperature and then were washed with sterile distilled water. The beads were conserved at 4 °C for a maximum of 24 h before performing the biodegradation tests. Thirty beads were then inoculated in MSM medium supplemented with crude oil and incubated as for the free consortium. In an attempt to improve the immobilized consortium efficiency, 0.5% sodium dodecyl sulphate (SDS) was also added, in a separate flask, to the MSM medium containing the entrapped consortium and incubated in the same conditions. At the end of the experiments after 5 months, beads in the medium without SDS were still intact. On the contrary, in the medium supplemented with SDS, apparent white and dark debris were observed, suggesting the disintegration of some beads.

### 2.7. Degradation of Crude Oil by the Free Isolates and the Bacterial Consortium

The degradation of the crude oil was monitored by gas chromatography-mass spectrometry (GC-MS). Residual crude oil was extracted with dichloromethane (DCM) from the MSM culture broth and the un-inoculated control. The solvent was mixed with the culture broth at a ratio of 1:2 (*v*/*v*). Sodium sulphate anhydrous (Na_2_SO_4_) was added to the collected organic layer in order to eliminate residual water. The crude oil was then filtered using a 0.2 µm screw cap microtube to avoid bacterial contamination.

The filtered samples were then diluted to 1:1000 with DCM. One microliter of each sample was then injected in the triple quadrupole Trace1300-TSQ quantum XLS ulta (Thermo Scientific, Bremen, Germany) instrument with helium as a carrier gas. The chromatographic separation was achieved using a Rxi-5Sil MS column by Restek (length 30 m, internal diameter 0.25 mm, film thickness 0.25 µm). The column temperature was set up at 45 °C with a hold time of 2 min and subsequent increase to 200 °C in ten minutes. The temperature increased up to 320 °C in 8 min with a final hold for 10 min. The full mass acquisition ranged from *m*/*z* 50–450. The obtained chromatograms were analyzed with Xcalibur (2.2) and the hydrocarbon compounds were identified using the NIST library database (2.0). The main compounds identified in crude oil are presented in Table 2. Differences among the different cultures were then evaluated considering the peak areas of the different compounds.

## 3. Results

### 3.1. Microbial Community

**Figure 1:**
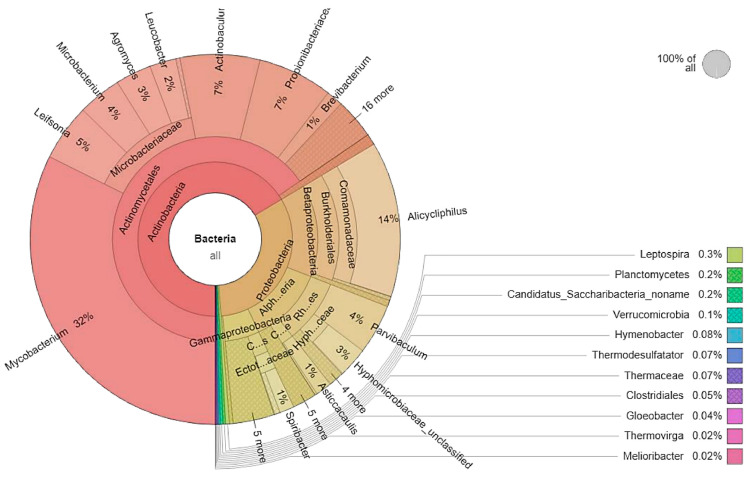
Taxonomic affiliation of micro-organisms in soil LF2.

The community profile analyzed with MetaPhlAn2 showed the large dominance of Bacteria in the LF2 soil sample, gathering 99.6% of the microbial population. Other micro-organisms were distributed among Archaea (0.1%), Eukaryota (0.02%), and viruses (0.32%). The taxonomic affiliation of micro-organisms in LF2 is presented in the Krona chart of Figure 1. Actinobacteria was the most abundant phylum (66.11%), followed by Proteobacteria (32.21%). Other phyla were detected at less than 1%, such as Firmicutes (0.05%).

Following the same trend, Actinobacteria (66.11%) was the most represented class. Betaproteobacteria (14.47%) was the second most abundant class, followed by Alphaproteobacteria (12.02%) and Gammaproteobacteria (5.39%). The other classes, such as Deinococci (0.07), were present at less than 1% relative abundance.

At the order level, Actinomycetales (65.15%) dominated the microbial community, followed by Burkholderiales (13.92%), Rhizobiales (8.33%), Chromatiales (1.80%), Caulobacterales (1.59%), and Oceanospirillales (1.05%).

At the family level, the top five most abundant taxa were Mycobacteriaceae (33.22%), Microbacteriaceae (14.51%), Comamonadaceae (13.58%), Actinomycetaceae (6.94%), and Propionibacteriaceae (6.52%). The main genera were represented by *Mycobacterium* (32.22%), followed by *Alicycliphilus* (13.55%) and *Actinobaculum* (6.94%). Genera *Microbacterium* (3.79%), *Gordonia* (0.67%), or *Pseudomonas* (0.21%) were present at lower relative abundances.

### 3.2. Isolation and Identification of Bacteria

In total, 37 bacterial strains were isolated from the landfarming-treated soil LF2. Twenty-eight isolates named LFS1 to LFS28 were obtained by direct plating of the soil solution, while nine strains, namely LFG1 to LFG9, were obtained from the diesel-enrichment culture. In the case of the auto mechanic workshop soil SGP, three bacteria belonging to the genus *Pseudomonas* were isolated and designated as SGPP1, SGPP2, and SGPP3. Specifically, strain SGPP3 presented a red pigmentation when plated on LB agar medium.

Twelve strains were randomly selected and identified by 16S rRNA gene sequencing (Table 3).

The results show that half of the isolates identified, namely strains *Bacillus wiedmannii* LFS26, *Bacillus paramycoides* LFS28, *Ochrobactrum haematophilum* LFG2, *Staphylococcus saprophyticus* LFG3, *Bacillus cereus* LFG4, *Ochrobactrum* sp. LFG6, and *Pseudomonas aeruginosa* SGPP3, were potentially pathogenic and therefore excluded to form consortia. Strains LFS6 (*Bacillus safensis*) and LFS15 (*Bacillus swezeyi*) belong to the *Bacillus* genus while strain LFG9 was assigned to species *Shinella zoogloeoides*. Lastly, strains SGPP1 and SGPP2 were assigned to the same species *Pseudomonas guguanensis*.

### 3.3. Biosurfactant Production

Based on the results of the molecular identification, strains *Bacillus safensis* LFS6, *Bacillus swezeyi* LFS15, *Shinella zoogloeoides* LFG9, and *Pseudomonas guguanensis* SGPP2 were selected for biosurfactant production assays. The tests included oil-coated agar and emulsification index.

**Figure 2:**
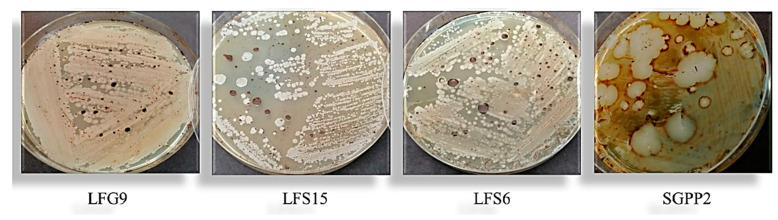
Oil-coated agar test of isolates *Shinella zoogloeoides* LFG9, *Bacillus swezeyi* LFS15, *Bacillus safensis* LFS6, and *Pseudomonas guguanensis* SGPP2 with crude oil as hydrocarbon substrate.

Strain SGPP2 alone presented positive biosurfactant activity on the LB agar plate with a halo around the colonies. The other strains showed no biosurfactant activity regarding the oil agar method (Figure 2).

**Figure 3:**
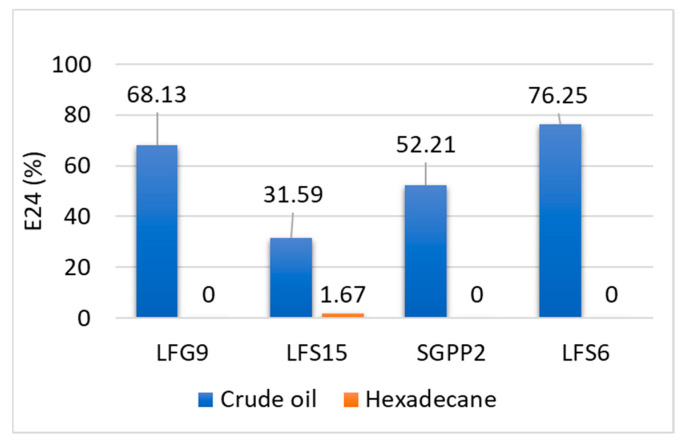
Emulsification index E_24_ of bacterial isolates *Shinella zoogloeoides* LFG9, *Bacillus swezeyi* LFS15, *Pseudomonas guguanensis* SGPP2, and *Bacillus safensis* LFS6 against crude oil and hexadecane.

On the contrary, all strains could emulsify crude oil with an E24 emulsification index ranging from 31.6% for LFS15 to 76.3% for LFS6. However, only isolate LFS15 could emulsify hexadecane with a low emulsification index, i.e., 1.7% (Figure 3).

These results show that the tested strains are biosurfactant producers.

### 3.4. Investigation of Crude Oil Degradation in Microcosms

**Figure 4:**
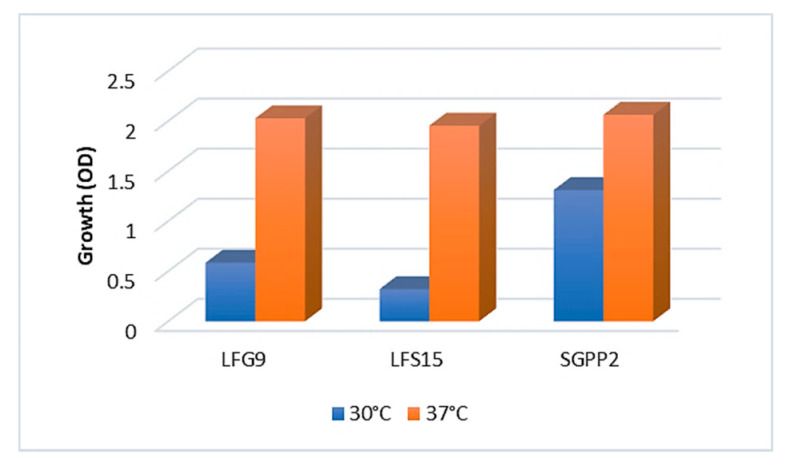
Growth of bacterial strains at 30 °C and 37 °C after 6 days. Bars represent the difference between the final and initial values of the optical densities (600 nm). LFG9: *Shinella zoogloeoides*; LFS15: *Bacillus swezeyi*; SGPP2: *Pseudomonas guguanensis*.

Although strain LFS6 exhibited the highest emulsification index against crude oil, this isolate presented an antagonism effect when grown with the other strains and was therefore excluded from the consortium formation. As a result, isolates *Bacillus swezeyi* LFS15, *Shinella zoogloeoides* LFG9, and *Pseudomonas guguanensis* SGPP2 were chosen to form the consortium COG1. As shown in Figure 4, the three strains exhibited the best growth when cultivated at 37 °C compared to 30 °C. Growth significantly increased by 3.5-fold, 6.1-fold, and 1.6-fold for strains LFG9, LFS15, and SGPP2, respectively, at 37 °C compared to 30 °C. Such results were expected as the bacterial strains were isolated from soils originating in a country with AW climate, according to the Köppen classification, which corresponds to an equatorial climate [22]. Therefore, the consortium as well as the individual strains were investigated for their ability to degrade crude oil at 37 °C.

**Figure 5:**
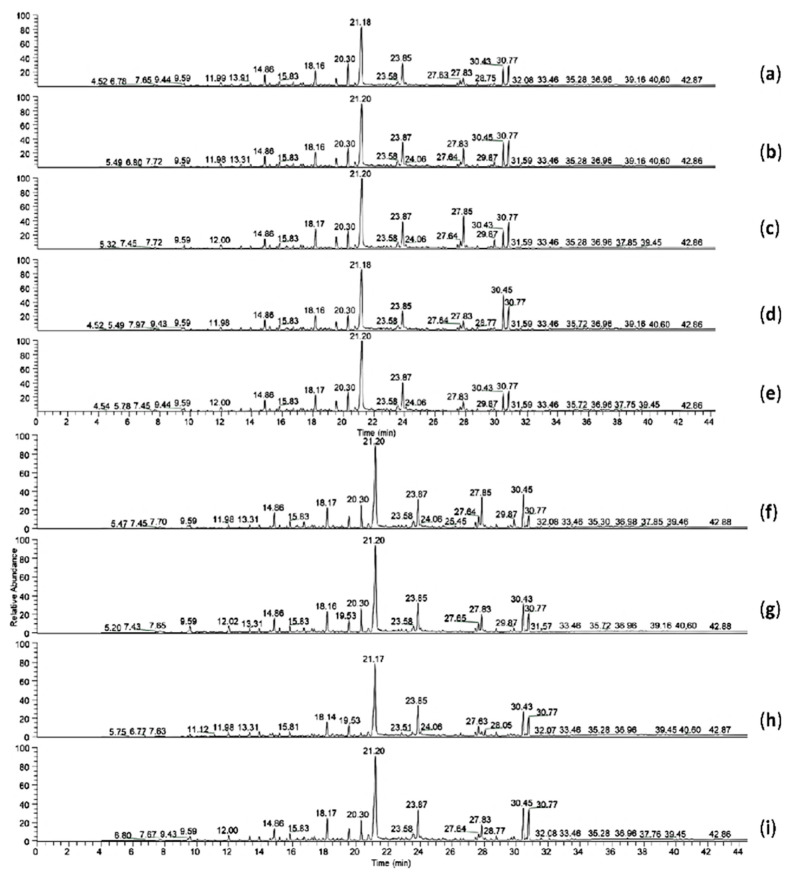
GC-MS chromatograms of crude oil degradation caused by (**a**) control; (**b**) *Bacillus swezeyi* LFS15 after 14 days; (**c**) *Bacillus swezeyi* LFS15 after 8 months; (**d**) *Shinella zoogloeoides* LFG9 after 14 days; (**e**) *Shinella zoogloeoides* LFG9 after 8 months; (**f**) *Pseudomonas guguanensis* SGPP2 after 14 days; (**g**) free consortium COG1 after 5 months; (**h**) entrapped consortium COG1E after 5 months; (**i**) entrapped consortium supplemented with SDS as COG1ES after 5 months.

After 14 days of treatment, phthalic anhydride (C_8_H_4_O_3_) was completely degraded by the three individual isolates. However, some differences were observed regarding the other crude oil components (Figure 5). A reduction of nonanoic acid and octadecanoic acid by 19.7% and 25.6%, respectively, compared to the control, was registered with strain LFG9, while these compounds were reduced by 12.2% and 7.3%, respectively, in the SGPP2 culture. Even lower degradation rates were obtained with strain LFS15, with a 10.3% and 1.9% reduction for nonanoic acid and octadecanoic acid, respectively. Additionally, n-decanoic acid, a coelution of benzofuran and phthalate, and an aromatic compound with a lateral unsaturated chain also decreased in strain LFG9 culture by 5.9%, 10.7%, and 13.76%, respectively, while these compounds increased in SGPP2 and LFS15 cultures. In the same way, tetradecanoic acid and phenol with amino group concentrations were reduced in LFG9 (5.7% and 12.3%, respectively, compared to the control) and LFS15 (6% and 7.5%, respectively, compared to the control) cultures, but increased in SGPP2 culture. Moreover, 2,4-bis(1,1-dimethylethyl)-phenol (C_14_H_22_O) was the only compound that increased in the LFG9 culture, while its concentration in SGPP2 and LFS15 decreased by 11.5% and 20%, respectively, compared to the control. The concentrations of the other compounds, including dodecanoic acid (C_12_H_24_O_2_), pentadecanoic acid (C_15_H_30_O_2_), n-hexadecanoic acid (C_16_H_32_O_2_), and 13-Docosenamide, (Z)-(C_22_H_43_NO), increased in all the three cultures at the end of the 14-day incubation period.

**Figure 6:**
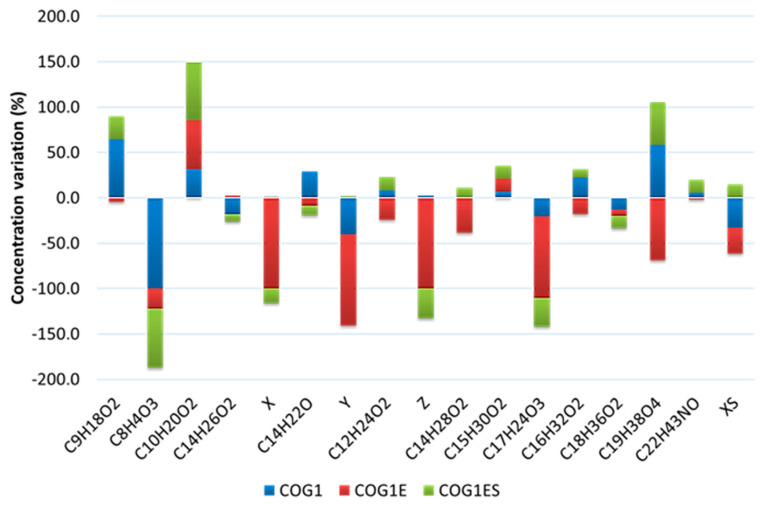
Variations of the main crude oil component concentrations expressed as relative quantity, induced by the free (COG1) consortium and entrapped (COG1E) consortium, as well as the entrapped consortium supplemented with SDS (COG1ES). Positive and negative numbers indicate an increase and degradation of the compound after the treatment, respectively. Numbers are given in percentages compared to the control. Formulas refer to: nonanoic acid (C_9_H_18_O_2_); phthalic anhydride (C_8_H_4_O_3_); n-decanoic acid (C_10_H_20_O_2_); 2,4,7,9-tetramethyl-5-decyn-4,7-diol (C_14_H_26_O_2_); unresolved coelution of benzofuran and phthalate (X); 2,4-bis(1,1-dimethylethyl)-phenol (C_14_H_22_O); unresolved aromatic compound with lateral unsaturated chain (Y); dodecanoic acid (C_12_H_24_O_2_); phenol with amino group (Z); tetradecanoic acid (C_14_H_28_O_2_); pentadecanoic acid (C_15_H_30_O_2_); 7,9-Di-tert-butyl-1-oxaspiro(4,5)deca-6,9-diene-2,8-dione (C_17_H_24_O_3_); n-hexadecanoic acid (C_16_H_32_O_2_); octadecanoic acid (C_18_H_36_O_2_); Hexadecanoic acid, 2-hydroxy-1-(hydroxymethyl)ethyl ester (C_19_H_38_O_4_); 13-Docosenamide, (Z)-(C_22_H_43_NO); unresolved unsaturated long-chain alkene (XS).

When the single strains were incubated for a longer period, i.e., 8 months, they performed similarly to the free consortium COG1 when incubated for 5 months, regarding most of the crude oil components (Figure 5). It was observed that phthalic anhydride was the only compound also fully degraded by COG1. However, a higher biodegradation performance was registered with COG1 for an aromatic compound with a lateral unsaturated chain and octadecanoic acid, which decreased by 40.6% and 13%, respectively, when instead, these compounds increased in LFG9 and LFS15 cultures in 8 months. Another pattern was detected for the entrapped consortium (COG1E). Although COG1E induced a decrease in the concentration of phthalic anhydride in crude oil (22% compared to the control), the compound did not disappear from the culture. Instead, in COG1E culture, a coelution of benzofuran and phthalate and an aromatic compound with a lateral unsaturated chain, as well as phenol with amino group, disappeared compared to the control culture, indicating their complete degradation unlike in the COG1 culture (Figure 6). The addition of sodium dodecyl sulphate did not lead to a particular improvement of hydrocarbon degradation as no compound fully disappeared in the treatment COG1ES. However, COG1ES treatment showed a higher decrease (65.1% reduction compared to the control) of phthalic anhydride compared to COG1E. In the same way, the reduction of the octadecanoic acid (C18H36O2) concentration in COG1E (6.7% compared to the control) was doubled in COG1ES (13.8%) (Figure 6). COG1E particularly presented a reduction of nonanoic acid (C9H18O2); dodecanoic acid (C_12_H_24_O_2_); tetradecanoic acid (C_14_H_28_O_2_); n-hexadecanoic acid (C_16_H_32_O_2_); hexadecanoic acid 2-hydroxy-1-(hydroxymethyl) ethyl ester, also known as glyceryl palmitate (C_19_H_38_O_4_); and (Z)-13-docosenamide by 4.6%, 24.4%, 38.6%, 18%, 68.9%, and 1.6%, respectively, compared to the control culture. Meanwhile, the concentration of these compounds increased in COG1 and COG1ES treatments (Figure 6).

## 4. Discussion

This work investigated the hydrocarbon potential of bacteria isolated from soils in the Republic of Congo and their efficiency as a consortium. Furthermore, the impact of alginate-bead-entrapment on the consortium effectiveness is determined. Here, we isolated bacterial strains from the landfarming-treated soil LF2 and the auto mechanic workshop soil SGP. These two types of soil are valuable sources for the isolation of hydrocarbon-degrading bacterial communities. Landfarming soil contaminated with petroleum waste has been shown to be a reservoir of microbial communities holding genes involved in the degradation of oil compounds [23]. Additionally, previous studies have reported the isolation of bacterial strains from diverse genera with the ability to degrade petroleum hydrocarbons [10,24,25,26]. Particularly, the investigation of the microbial communities in SGP and their functional profiles will be communicated in another study. The high throughput sequencing of the landfarming soil in the present work indicated the presence of bacterial hydrocarbon-degrading groups, such as Actinomycetales and Burkholderiales [27]. In accordance with our results, Actinomycetales were also reported as the dominant sub-group in Actinobacteria taxon in landfarming-treated soils [28,29]. The presence of these orders in addition to other taxonomic groups associated with hydrocarbon contamination, such as Gammaproteobacteria, Microbacteriaceae, *Mycobacterium*, *Gordonia*, and *Pseudomonas* [14,27,29], indicate the positive potential of the bacterial communities in LF2 for bioremediation strategies. In this context, bacterial strains were isolated from LF2 in order to build a consortium. The members of the consortium were selected based on their hydrocarbon degradation and biosurfactant production abilities. The use of bacterial consortia for the remediation of petroleum hydrocarbons have expanded in recent years with successful reports [1,19,24,30]. Microbial consortia particularly offer greater advantages for hydrocarbon degradation compared to single strains, owing to the presence of more diverse metabolic functionalities [6].

Although Firmicutes members were found at particularly low abundance (0.05%) in our work, most of the isolates identified in the landfarming soil were *Bacillus* species, namely *Bacillus safensis*, *Bacillus swezeyi*, *Bacillus wiedmannii*, *Bacillus paramycoides*, and *Bacillus cereus*. This genus is often encountered in petroleum-contaminated sites [1,27] and its members are known for their capacity to degrade complex hydrocarbons [6,7,19]. *Bacillus* species are also considered valuable candidates for petroleum recovery due to endospore formation. Endospores confer a strong tolerance to high concentrations of hydrocarbons as well as a great resistance to abiotic factor fluctuations and lasting survival [7,31]. A *Shinella zoogloeoides* strain, LFG9, was also isolated from LF2. The genus *Shinella* was described in 2006 [32]. *Shinella* strains have been reported for the degradation of xenobiotics, such as nicotine [33], 4-aminobenzenesulfonate [34], or 1H-1,2,4-triazole (TZ) [35], and the assimilation of some compounds, such as pyridine [36] and lead [37]. However, this genus has not been extensively studied for its hydrocarbon remediation potential. Particularly, only a few studies have reported the degradation of petroleum compounds, such as anthracene [38], by *Shinella zoogloeoides*. This species is therefore worth investigating for hydrocarbon bioremediation. Other isolates obtained in this study belonged to *Pseudomonas*, a genus renowned for its hydrocarbon degradation capabilities, also via the synthesis of biosurfactants [9,12,39]. Particularly, we report the isolation of *Pseudomonas guguanensis* strain SGPP2. *Pseudomonas guguanensis* has been proposed as novel species by Ref. [40]. The abilities of *Pseudomonas guguanensis* in oil bioremediation are discussed in the literature with promising rates of total petroleum hydrocarbon depletion in crude-oil-contaminated soil [41], as well as hexadecane degradation [42] and hydrocarbon emulsification activity (i.e., hexadecane, diesel, kerosene, and crude oil) [42]. In our study, strains *Bacillus swezeyi* LFS15, *Shinella zoogloeoides* LFG9, and *Pseudomonas guguanensis* SGPP2 were selected to build a consortium. These strains exhibited emulsification activity and were able to degrade or reduce crude oil components, which indicated their hydrocarbon degradation capabilities. *Shinella zoogloeoides* LFG9 was able to emulsify crude oil at a favorable emulsification percentage (68.1%). To the best of our knowledge, this is the first time that this species is reported for its crude oil emulsification ability. *Pseudomonas guguanensis* SGPP2 also displayed a positive emulsification index against crude oil (52.2%). However, the strain did not emulsify hexadecane as shown in another study [42]. In fact, the authors suggested that the resulting fatty acids from hexadecane degradation were used by their *Pseudomonas guguanensis* strain to synthesize rhamnolipids, which in turn broke down hexadecane, therefore creating a degradation loop [42]. This suggests that our strain *Pseudomonas guguanensis* SGPP2 might produce emulsifiers with different structure and characteristics and display a specific pattern for biosurfactant production worth targeting for deeper investigation and comparative studies. *Bacillus swezeyi* LFS15 also presented an emulsification ability towards crude oil, although at lower percentage compared to the other strains. Emulsification towards hexadecane was also observed. *Bacillus* members are great biosurfactant producers [2,10], such as the species *Bacillus licheniformis* to which *Bacillus swezeyi* is closely related [43].

The hydrocarbon degradation ability of the strains and, mostly, the efficiency of their consortium was assessed in microcosms with crude oil as the hydrocarbon source. GC-MS analysis of the blank culture revealed that the crude oil in this study was mainly composed of saturated fatty acids (C9-C10 and C12-C19) as well as aromatics, including those not fully resolved and identified (benzofuran, phthalic acid derivatives, and an unknown aromatic compound with lateral unsaturated chain) and an unknown unsaturated alkene. Ref. [8] found similar compounds in the composition of the crude oil used in their study.

The three isolates as single cultures were able to fully remove phthalic anhydride from crude oil. In fact, phthalic anhydride was the only crude oil component fully degraded at the same time in all microcosms, either single strains or a free and immobilized consortium. This compound has been reported as an intermediate metabolite in the biodegradation of phenanthrene by a consortium of *Bacillus* and *Acidovorax* strains [30]. In addition to that, a reduction of other crude oil compounds found in this study was registered for all single strains. Overall, strain *Shinella zoogloeoides* LFG9 performed better than the two other isolates, *Pseudomonas guguanensis* SGPP2 and *Bacillus swezeyi* LFS15, after 14 days of incubation. An increase in the incubation time to 8 months induced higher degradation of certain compounds in the LFG9 culture, mainly phenolic components found in crude oil (phenol, 2,4-bis(1,1-dimethylethyl); phenol with amino group). At the same time, other compounds (13-Docosenamide, (Z)-; unknown unsaturated long-chain alkene) not previously degraded (after 14 days) were reduced under the control culture concentration. These results are consistent with a previous report suggesting a preferential degradation of certain compounds by bacteria and with time [5].

The co-culture of the three strains as a free consortium (COG1) showed a slight enhancement of crude oil degradation. It is expected to observe an increase of contaminant biodegradation by a consortium compared to single strains when no antagonism is detected. As an example, Ref. [44] reported a significant improvement of pyrene degradation by their consortium of *Klebsiella pneumoniae* and *Pseudomonas aeruginosa* compared to the single strains. In this study, COG1 showed a particular improvement of biodegradation for the aromatic compound with a lateral unsaturated chain and octadecanoic acid. A reduction of 33% for the unsaturated long-chain alkene compared to the control was also registered, as well as an increase of all the fatty acid concentrations, except octadecanoic acid. Ref. [21] hypothesized that the biodegradation of the high molecular weight fractions of crude oil in their study resulted in the accumulation of hydrocarbons with a lower molecular weight. In the same way, ref. [19] reported the formation of esters and acids as intermediate metabolites in crude oil biodegradation caused by a consortium of *Bacillus pumilus* and *Bacillus cereus*. Ref. [8] also reported the accumulation of acid groups after 10 days of crude oil degradation caused by their wheat-bran-immobilized consortium (*Oceanobacillus* sp., *Nesiotobacter* sp., *Ruegeria* sp., *Photobacterium* sp., *Enterobacter* sp., *Haererehalobacter* sp., *Exiguobacterium* sp., *Acinetobacter* sp., and *Pseudoalteromonas* sp.) as an indication of hydrocarbon compound oxidation. However, in our study, we assumed that unsaturated and aromatic compound degradation resulted in their transformation into fatty acids and therefore the increase of the latter would require more investigations.

Immobilization changed the biodegradation pattern of the bacterial consortium and positively affected its efficiency. Indeed, the immobilized consortium (COG1E) exhibited a broader degradation of aromatic compounds. This included the degradation of the most recalcitrant compounds found in this study, such as benzofuran, which was completely degraded. Previous studies reported biodegradation improvement after immobilization. For instance, ref. [21] reported a 31.9% and 1.9% increase in normal alkanes and PAH degradation, respectively, caused by their consortium comprising five strains, after immobilization. Similarly, ref. [1] registered a 2.5% increase in TPH degradation in crude-oil-contaminated soil after the immobilization of *Pseudomonas* sp. and the *Bacillus amyloliquefaciens* consortium on bentonite–alginate beads, as compared to the free consortium. This can be explained by the fact that immobilization provides room for a better tolerance and resilience for hydrocarbon toxicity caused by micro-organisms. Moreover, the porous matrix allows metabolite exchange with the environment and a greater microbial growth compared to free cells. As a result, degradation performance is improved [8]. Some authors pointed out the fact that biomass transfer is promoted by bead structure, which contributes to the adsorption of the immobilized biomass on the beads’ surface. Consequently, the contact with contaminants and their subsequent uptake is enhanced [44].

Biodegradation can further be improved not only by addition of nutrients, such as nitrogen and phosphorus [8,13], but also by the use of surfactants. The latter intervene in crude oil emulsification and can be microbial-growth-promoting factors [2]. Tween 80 is one of the model surfactants used to enhance crude oil degradation by the bacterial consortium. This non-ionic surfactant can serve as a carbon source for the bacterial consortium, therefore promoting microbial growth. However, high concentrations can be damaging for microbial biomass [2]. Likewise, ref. [12] showed an increased abundance of hydrocarbon-degrading bacteria when applying several dispersants (i.e., Superdispersant 25, Slickgone NS) for oil degradation in microcosms. Another surfactant that has also been investigated to improve hydrocarbon degradation is SDS [2]. In their study, ref. [2] showed a slight increase of 4% crude oil biodegradation with the addition of SDS at a concentration of 1 CMC (600 mg/L).

In the present study, although a slight decrease of certain compounds was observed, the addition of SDS (COG1ES) did not improve crude oil degradation compared to the use of the entrapped consortium alone. On the contrary, it seemed that SDS inhibited its efficiency. It can be hypothesized that the concentration of SDS used in this study was too high, thus having a toxic effect on bacterial strains. This could have been coupled with a change in the pH medium, which became slightly unfavorable for crude oil degradation. The toxicity of SDS on microbial cells at certain concentrations and its oil degradation inhibitory effect have been documented in previous studies [2]. Phenomena of hydrocarbon biodegradation inhibition by SDS were observed by [45]. Moreover, ref. [21] showed that although immobilization improved the tolerance of micro-organisms for pH variations in their study, those variations still slightly affected crude oil degradation at 4.3% when the pH varied between 6 and 9.

Another hypothesis would be the preferential use of SDS as a carbon source by bacteria instead of crude oil in this study, therefore inducing a limited degradation of crude oil. Similar suggestions were made by Ref. [45]. According to the authors, the preferential use of surfactants by HAP degraders was responsible for the inhibition of hydrocarbon biodegradation. According to Ref. [46], SDS can be used as a growth substrate for micro-organisms during hydrocarbon biodegradation and further lead to reduced hydrocarbon utilization.

Another possibility is that bacteria found a more accessible alternative source of carbon in the polysaccharides released from alginate. Indeed, the disaggregation of the beads, suggested by the presence of white debris in COG1ES medium, seems possible. SDS can capture the calcium ions (Ca^2+^) from the alginate gel, owing to its capacity to interact with metal ions in solution, especially Ca^2+^ [47,48]. In this case, the bead structure is decomposed, accompanied with the formation of calcium dodecyl sulfate (Ca(DS)_2_), which precipitates that it might correspond to the black debris in the medium.

Based on these results it appears clear that alginate-bead-entrapment provided a safe environment that promoted synergetic interactions between strains *Bacillus swezeyi* LFS15, *Shinella zoogloeoides* LFG9, and *Pseudomonas guguanensis* SGPP2 in the consortium for crude oil degradation. The efficiency of the entrapped consortium can be improved with the utilization of surface-active agents. However, the supplementation of biosurfactants is preferable to the addition of synthetic surfactants, such as SDS. Taking as example the study by Ref. [8], who showed compatibility between rhamnolipids and *Pseudomonas* genus for oil degradation improvement, the supplementation of biosurfactants produced by one of the consortium members represents a better alternative to enhance the efficiency of COG1 for further investigation. Additionally, strain *Shinella zoogloeoides* LFG9, which best performed among the three strains, is worth studying in association with other strains for enhanced oil recovery caused by the alginate-bead-entrapped bacterial consortium.

## 5. Conclusions

In this work, the investigation of the microbial community structure of the landfarming-treated soil LF2 revealed the presence of hydrocarbon-degrading bacterial genera. Among the nine strains identified from soil LF2, two isolates belonging to species *Bacillus swezeyi* and *Shinella zoogloeoides*, LFG9 and LFS15, respectively, were associated with the strain *Pseudomonas guguanensis* SGPP2 to form the consortium COG1. The bacterial consortium that immobilized in alginate beads performed better than the free consortium in crude oil degradation. Immobilization provided protection and better tolerance of crude oil toxicity to bacterial cells, therefore improving the biodegradation of aliphatic, branched, and aromatic compounds. The inhibition of the biodegradation process caused by the addition of SDS indicated that entrapment was the main factor responsible for the enhanced crude oil removal. This technique is therefore suitable for further investigation of hydrocarbon bioremediation using the bacterial members of the COG1 consortium. Especially, strain *Shinella zoogloeoides* LFG9, which best performed in crude oil degradation among the three isolates, is a valuable candidate for enhanced oil recovery by means of immobilized bacterial consortia.

## Figures and Tables

**Table 1:** Characteristics of soil LF2.

Parameters	Values
Clay (%)	13.70 ± 2
Slit (%)	20.92 ± 3.93
Sand (%)	65.35 ± 2.21
C (g/kg)	60.93
N (g/kg)	2.01
P (g/kg)	0.1
Fe (mg/kg)	9500
K (mg/kg)	982
Ca (mg/kg)	2020
Mg (mg/kg)	5580
Mn (mg/kg)	145
pH	6.11
TPH (g/kg)	355

**Table 2:** Main compounds identified in crude oil by GC-MS. Rt indicates the retention time expressed in minutes.

Compounds	Rt	Compounds	Rt
Nonanoic acid	12.00	Tetradecanoic acid	18.16
Phthalic anhydride	12.68	Pentadecanoic acid	19.50
n-Decanoic acid	13.31	7.9-Di-tert-butyl-1-oxaspiro (4.5) deca-6.9-diene-2.8-dione	20.30
2.4.7.9-Tetramethyl-5-decyn-4.7-diol	13.91	n-Hexadecanoic acid	21.06
Coelution of benzofuran and phthalate	14.86	Octadecanoic acid	23.85
2.4-bis(1.1-dimethylethyl)-phenol	15.20	Hexadecanoic acid. 2-hydroxy-1-(hydroxymethyl)ethyl ester	27.82
Unknown aromatic compound with lateral unsaturated chain	15.61	13-Docosenamide. (Z)-	30.43
Dodecanoic acid	15.81	Unknown unsaturated long chain alkene	30.77
Unknown phenolic compound with amino group	16.75		

**Table 3:** Bacterial strain identification.

Strain	Identification	GenBank Accession N
LFS6	*Bacillus safensis*	MT672419.1
LFS15	*Bacillus swezeyi*	MT672420.1
LFS26	*Bacillus wiedmannii*	MT672421.1
LFS28	*Bacillus paramycoides*	MT672336.1
LFG2	*Ochrobactrum haematophilum*	MT672422.1
LFG3	*Staphylococcus saprophyticus*	MT672338.1
LFG4	*Bacillus cereus*	MT672423.1
LFG6	*Ochrobactrum* sp.	MT672337.1
LFG9	*Shinella zoogloeoides*	MT672424.1
SGPP1	*Pseudomonas guguanensis*	MT672417.1
SGPP2	*Pseudomonas guguanensis*	MT672418.1
SGPP3	*Pseudomonas aeruginosa*	MT672425.1

## Data Availability

Metagenomics data were deposited in NCBI Sequence Read Archive (SRA) under the accession number PRJNA853725.
